# Iron Oxide Nanozyme for Magnetothermal Activation of ER-TRPV1 Enables Efficient Intravesical Therapy of Bladder Cancer

**DOI:** 10.3390/ijms27167475

**Published:** 2026-08-21

**Authors:** Galong Li, Dongyan Li, Bin Lan, Yuanyuan Cheng, Hongxia Liang, Wenli Zhang, Xiaopan Xu, Hongbing Lu

**Affiliations:** 1 School of Biomedical Engineering, Fourth Military Medical University, Xi’an 710032, China; ligalong@fmmu.edu.cn (G.L.);; 2 Medical Innovation Center, Fourth Military Medical University, Xi’an 710032, China; 3 College of Life Sciences, Northwest University, Xi’an 710069, China

**Keywords:** bladder cancer, intravesical therapy, magnetic hyperthermia therapy, TRPV1, FGFR3

## Abstract

Drug-mediated intravesical therapy is a widely used treatment approach for bladder cancer (BCa), which is limited by poor tumor accumulation and the toxicity of drugs. Herein, a facile approach utilizing magnetic hyperthermia therapy (MHT) for BCa treatment is reported through intravesical instillation of tumor-targeting iron oxide nanoparticles (IONPs), which are coated with anti-FGFR3 antibodies for specific binding to FGFR3 overexpressed on BCa cells. The antibody coating significantly enhanced targeting ability and increased cancer cell uptake. Under an alternating magnetic field (AMF), the IONPs magnetothermally activated TRPV1 on the endoplasmic reticulum (ER) of BCa and induced Ca^2+^ overload in RT4 BCa cells. The magnetothermal treatment significantly upregulated the pro-apoptotic gene expression of Bax, Pro-caspase-3, and Cytc in RT4 cells, and downregulated the anti-apoptotic effector gene Bcl-2, ultimately causing augmented apoptosis in cancer cells. The application of intravesical FGFR3-targeting MHT successfully resulted in greater suppression of tumor growth and improved survival rate of mice, compared to the control, IONP, and AMF-treated groups. These results hold significant potential as a transformative modality toward BCa therapeutic application.

## 1. Introduction

Bladder cancer (BCa), the most prevalent urinary malignancy, remains a major and growing global health concern [1,2,3]. Non-muscle-invasive BCa makes up roughly 75%, and its current standard treatment is tumor resection followed by intravesical instillation of drugs for chemotherapy. However, the treatments remain less effective and have unresolved issues. The transurethral resection of the tumor is over- or under-resection, and there are even life-threatening issues and injury to normal tissues. Though frequent intravesical instillation of chemical drugs can kill unresected tumors [4,5,6], the treatment shows less effective therapeutic outcomes, owing to the short retention time of drugs in the bladder and poor tumor enrichment efficacy of drugs, along with toxicity to normal bladder tissues and drug resistance [7,8,9,10]. Therefore, there is a general recognition of the urgent need for developing a targeted and highly efficient intravesical therapeutic method.

To increase the drug enrichment rate in the tumor after intravesical instillation, driving intra-bladder diffusion of drugs that were modified with tumor-targeting molecules remains the most promising strategy for improving antitumor efficacy. The driving force is key to increasing the diffusion of targeted drugs, thus enabling them to specifically bind and kill tumor cells [11,12,13,14,15,16,17,18,19,20,21,22,23,24,25,26]. Studies have shown that various driving forces promote drug diffusion in the bladder [11]. For example, urease-coated radiolabeled mesoporous silica nanobots catalyzed the production of gas to enhance diffusion capability in urine, resulting in efficient accumulation and penetration at the tumor site and BCa therapy [13]. Nanoparticle-mediated thermal effect under NIR-II light has also been used as a driving force to endow nanoparticles with enhanced motion in urine and penetration capability in tumor tissue, but NIR light penetration depth limits its application in orthotopic BCa [27]. The thermal effect produced by magnetic nanoparticles under the action of an alternating magnetic field (AMF) has been shown to be effective at driving nanoparticles’ motion. It is expected to facilitate the delivery and accumulation of nanoparticles at the tumor site.

The magnetothermal effect has garnered extensive attention owing to its high effectiveness in magnetic hyperthermia therapy (MHT), promotion of nanozyme-catalyzed reactions, and activation of heat-sensitive ion channels. Specifically, tumor MHT utilizes the heat generated by magnetic nanoparticles under AMF to kill heat-sensitive tumor cells while sparing surrounding normal cells [28]. The magnetothermal effect (>42 °C) has also been used to activate the heat-sensitive transient receptor potential vanilloid member 1 (TRPV1) ion channel overexpressed on cells, triggering Ca^2+^ influx and controlling cell activity [29]. The magnetothermal effect can remarkably enhance the peroxidase-like activity of magnetic iron oxide nanoparticles (IONPs), which have been demonstrated as nanozymes [30]. Notably, biocompatible superparamagnetic IONPs represent the most extensively studied nanodrugs, due to their excellent biocompatibility, easy surface functionalization, water dispersibility, and structural stability [31]. Researchers have been devoted to developing innovative antibody-conjugated IONPs for improving magnetothermal interference precision and efficacy [32,33,34]. Hence, there is a close connection between tumor-targeting IONPs and multimodal and efficient intravesical BCa therapy, emphasizing the significance of the synergistic effect induced by the magnetothermal effect.

TRPV1 exerts tumor-suppressive functions in BCa [35]. For example, activation of TRPV1 by the agonist (capsaicin, CAP) has been demonstrated to inhibit cancer growth and induce apoptosis of cancer cells, and TRPV1 emerges as an important therapeutic target in BCa [34,36,37]. The overexpression of TRPV1 on the endoplasmic reticulum (ER) in BCa cells results in intracellular Ca^2+^ overload, eliciting inter-organellar stress, ultimately inducing apoptosis [38,39]. Therefore, precisely activating TRPV1 on the ER is a potent approach to trigger inter-organellar crosstalk and improve therapeutic efficacy in BCa, yet it remains unexplored.

**Figure 1:**
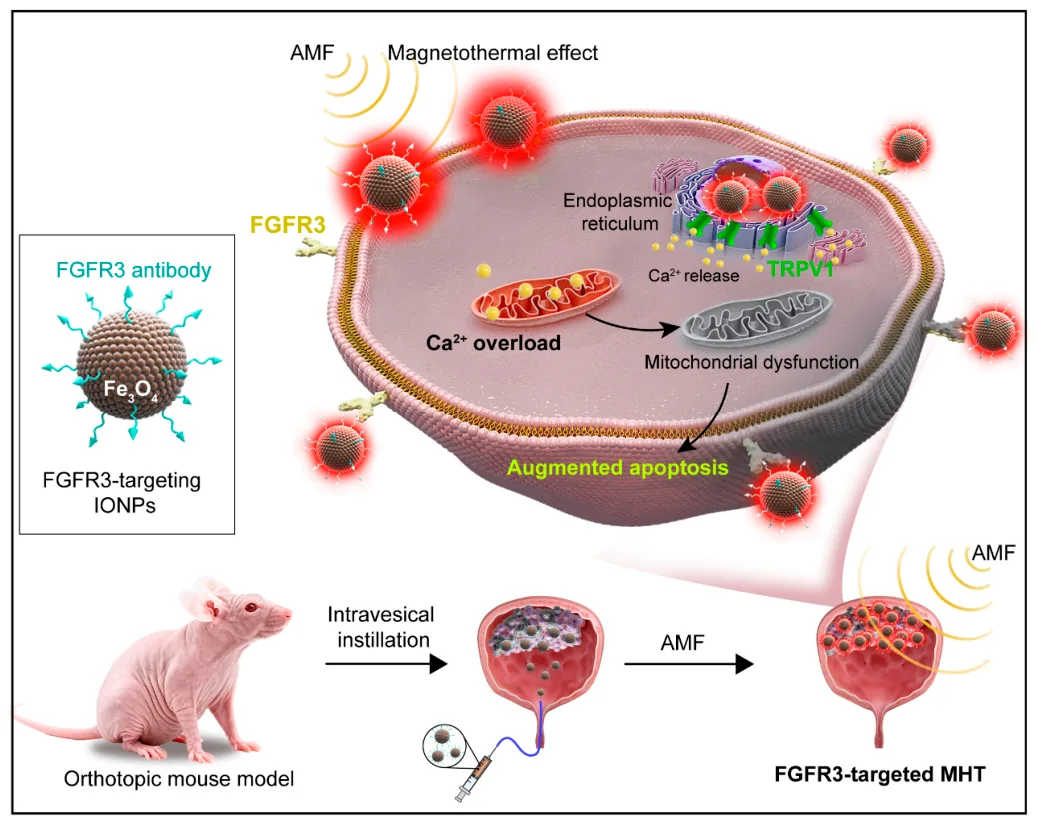
Schematic illustration of the ER-TRPV1-specific IONP-mediated MHT with augmented apoptosis caused by effective intracellular Ca^2+^ overload and heat-killing ability.

In this study, a facile and efficient strategy for intravesical tumor-targeting MHT of BCa is presented in which IONPs are utilized to activate ER-TRPV1, induce augmented apoptosis, and suppress tumor growth (as shown in Figure 1). Specifically, the IONPs were functionalized with antibodies against fibroblast growth factor receptor 3 (FGFR3), which is overexpressed on RT4 BCa cells. The anti-FGFR3 antibody-coated IONPs can effectively target the FGFR3 protein on the surface of RT4 cells after intravesical instillation and are subsequently internalized by cancer cells. Under an AMF, the intracellular IONPs exhibited quick heat-generating ability, which not only enhanced peroxidase-like activity for producing toxic reactive oxygen species (ROS) but also activated ER-TRPV1 for inducing cytoplasmic Ca^2+^ overload. The high Ca^2+^ concentration in the cancer cells leads to mitochondrial dysfunction and causes significant changes in the gene transcriptome. Combining Ca^2+^ overload with ROS-heat therapy achieved augmented apoptosis and significantly suppressed tumor growth in an orthotopic mouse model. Together, this ER-TRPV1-specific IONP-mediated MHT strategy with intracellular Ca^2+^ overload, ROS accumulation, and heat-killing effect provides a new strategy for potentiating the effectiveness of intravesical instillation therapy.

## 2. Results

### 2.1. Synthesis and Characterization of Nanoparticles

**Figure 2:**
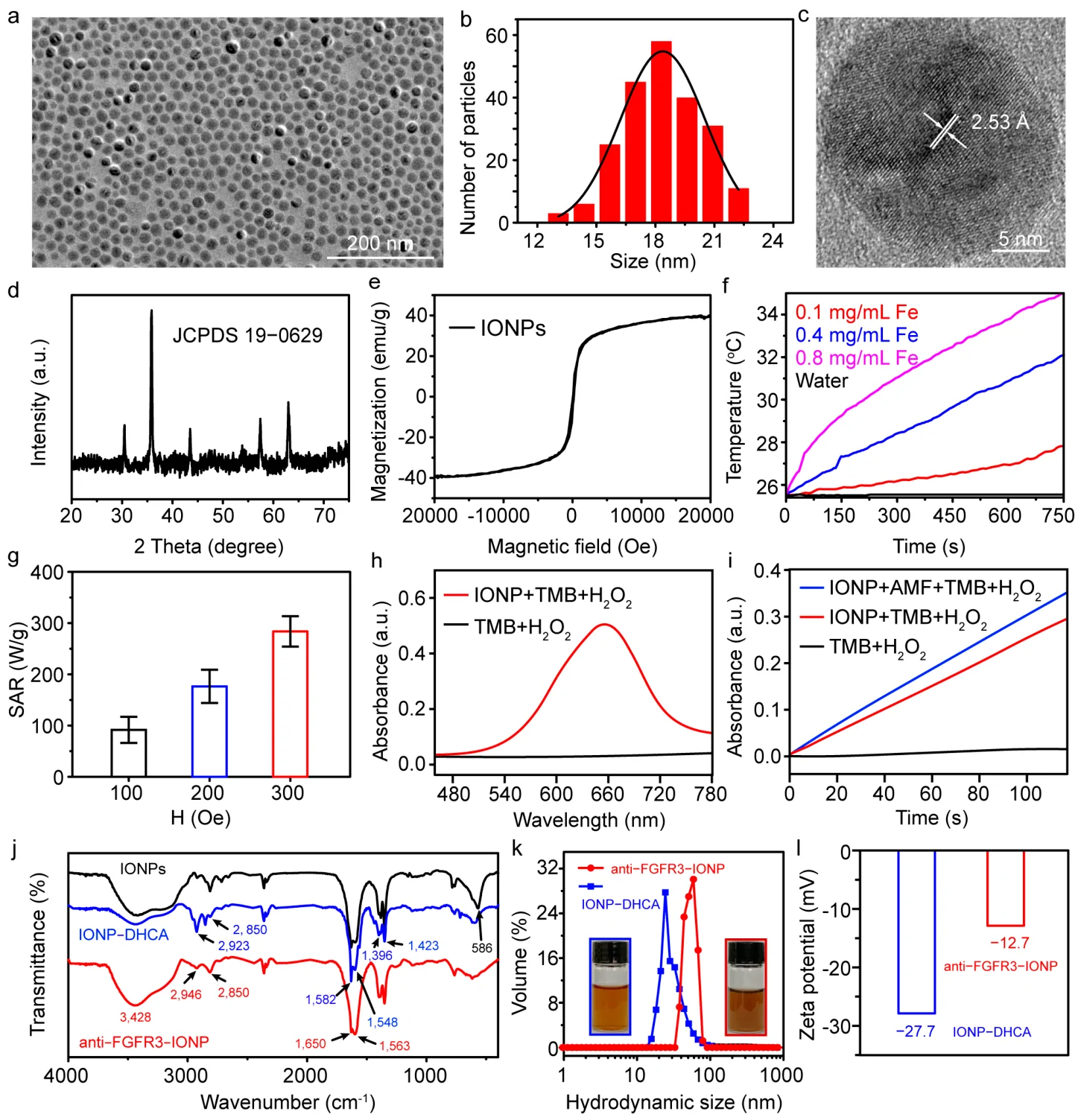
Characterization of IONPs. (**a**) TEM image, (**b**) particle size distribution profile, (**c**) HRTEM image, (**d**) X-ray diffraction pattern, (**e**) vibrating sample magnetometer measurement of IONPs. (**f**) Heating profiles of IONPs dispersed in DI water with different Fe concentrations under an AMF (315 kHz, 300 Oe). (**g**) SAR values of IONPs (Fe concentration: 0.4 mg/mL) under different AMF exposures (*f* = 315 kHz; H = 100, 200, and 300 Oe). (**h**) Typical absorption curves of the generated oxTMB at 652 nm and (**i**) time-dependent absorbance increase in oxTMB during IONP-based catalytic reaction. (**j**) FTIR spectra of IONPs, IONP-DHCA, and anti-FGFR3-IONP. (**k**) Hydrodynamic size distributions of IONP-DHCA and anti-FGFR3-IONP; insets are the corresponding photos. (**l**) Zeta potential of IONP-DHCA and anti-FGFR3-IONP. Data are displayed as mean ± SD.

Monodisperse IONPs with a uniform diameter were prepared via high-temperature thermal decomposition. Transmission electron microscopy (TEM) confirmed the uniform diameter (18.37 nm) and spherical morphology of the synthesized nanoparticles (Figure 2a,b). As shown in the high-resolution TEM (HRTEM) image presented in Figure 2c, the IONPs displayed a defined lattice fringe (∼2.53 Å), which can be attributed to the (311) plane of Fe_3_O_4_, suggesting the single-crystal nature of the IONPs. The X-ray powder diffraction pattern further confirmed the magnetite phase (JCPDS no.19-0629) and typical cubic spinel structure of the IONPs (Figure 2d). The vibrating sample magnetometer (VSM) measurement revealed that the IONPs exhibited a high magnetic saturation strength of 40.6 emu/g (Figure 2e). The hysteresis curves of IONPs exhibited no significant hysteresis, suggesting the unique superparamagnetic nature of IONPs. Therefore, the IONPs disperse well in water without spontaneous aggregation, which is beneficial to their biomedical applications.

The 3,4-dihydroxyhydrocinnamic acid (DHCA) was functionalized on IONPs by replacing the surface-bound oleic acid via a ligand exchange reaction; thereby, the IONP-DHCA showed colloidal stability. The magnetothermal performance of IONP-DHCA was subsequently evaluated using a magnetic induction heating system equipped with a thermocouple fiber. Figure 2f shows the temperature increase profiles of IONP-DHCA with Fe concentrations varying from 0 to 0.8 mg/mL under AMF exposure (*f* = 315 kHz; 300 Oe). The temperature of the IONP-containing solution (0.8 mg/mL) quickly increased from 25 °C to approximately 33 °C within 600 s when exposed to the AMF. The field-dependent SAR for IONPs is displayed in Figure 2g. With an increase in the amplitude from 100 to 300 Oe, the SAR of the IONPs varied from 93.5 to 290.7 W/g. In addition, the IONP-DHCA in the H_2_O_2_-containing buffer showed intrinsic peroxidase-like activity, which catalyzed the oxidation reaction of 3,3,5,5-tetramethylbenzidine (TMB) to a blue color (with maximum absorbance at 652 nm) and reactive oxygen species (ROS) (Figure 2h,i). Overall, these results demonstrated that the IONPs were capable of effectively generating heat for TRPV1 activation and MHT under AMF exposure.

The anti-FGFR3 antibodies were covalently conjugated to the surface of IONP-DHCA via an EDC/NHS coupling reaction. To further confirm this, we performed FTIR spectroscopy analysis on the IONPs, IONP-DHCA, and anti-FGFR3-IONPs (as shown in Figure 2j). The bands at 3428 cm^−1^ are attributed to the NH stretching vibrations of amines. The band at 1650 cm^−1^ is the Amide I band, attributed to C=O vibrations in amide groups. The band at 1563 cm is the Amide II band and is attributed to NH vibrations in the amide group, as reported in previous work [40]. The newly formed amide bonds between the carboxyl group of DHCA and the amine group of antibodies via an EDC/NHS reaction confirm that the anti-FGFR3 antibodies were covalently modified onto IONPs. In the following results and discussion, we simplify the abbreviation of anti-FGFR3-IONPs to IONPs. Dynamic light scattering (DLS) measurements showed consistent results. After antibody conjugation, the anti-FGFR3-IONPs exhibited an intact particle morphology and a hydrodynamic diameter of 66.56 nm, much higher than the 21.61 nm of IONP-DHCA (Figure S1 and Figure 2k). The hydrodynamic size of the anti-FGFR3-IONPs remained around 91.28 nm in artificial urine (Figure S2). The results confirm that the anti-FGFR3-IONP is robust against the harsh urinary environment. Simultaneously, the zeta potential of the anti-FGFR3-IONPs was −12.7 mV, higher than the 27.7 mV of IONP-DHCA (Figure 2l), indicating that the anti-FGFR3 antibodies were successfully functionalized on the surface of IONPs. We further evaluated the stability of the anti-FGFR3-IONPs after AMF exposure. The hydrodynamic diameter of the anti-FGFR3-IONPs remained stable (66.56 nm before vs 81.45 nm after AMF), with a PDI consistently below 0.2, indicating no significant aggregation (Figure S3a). Zeta potential measurements also showed no significant change (−12.7 mV before vs −14.3 mV after AMF) (Figure S3b), suggesting that our nanoparticle maintains excellent colloidal stability under AMF exposure.

### 2.2. Antitumor Activity of FGFR3-Targeting IONPs In Vitro

Before in vitro studies, the cytotoxicity of IONPs at various Fe concentrations (from 0 to 100 μg/mL) in RT4 cells was examined via the CCK-8 assay. As shown in the results depicted in Figure S4, the detected cell viability remained above 95% after 24 h of incubation with IONPs at Fe concentrations as high as 100 μg/mL. This suggested that the synthesized IONPs have excellent biocompatibility, providing a reliable experimental basis for follow-up in vitro and in vivo therapeutic studies.

**Figure 3:**
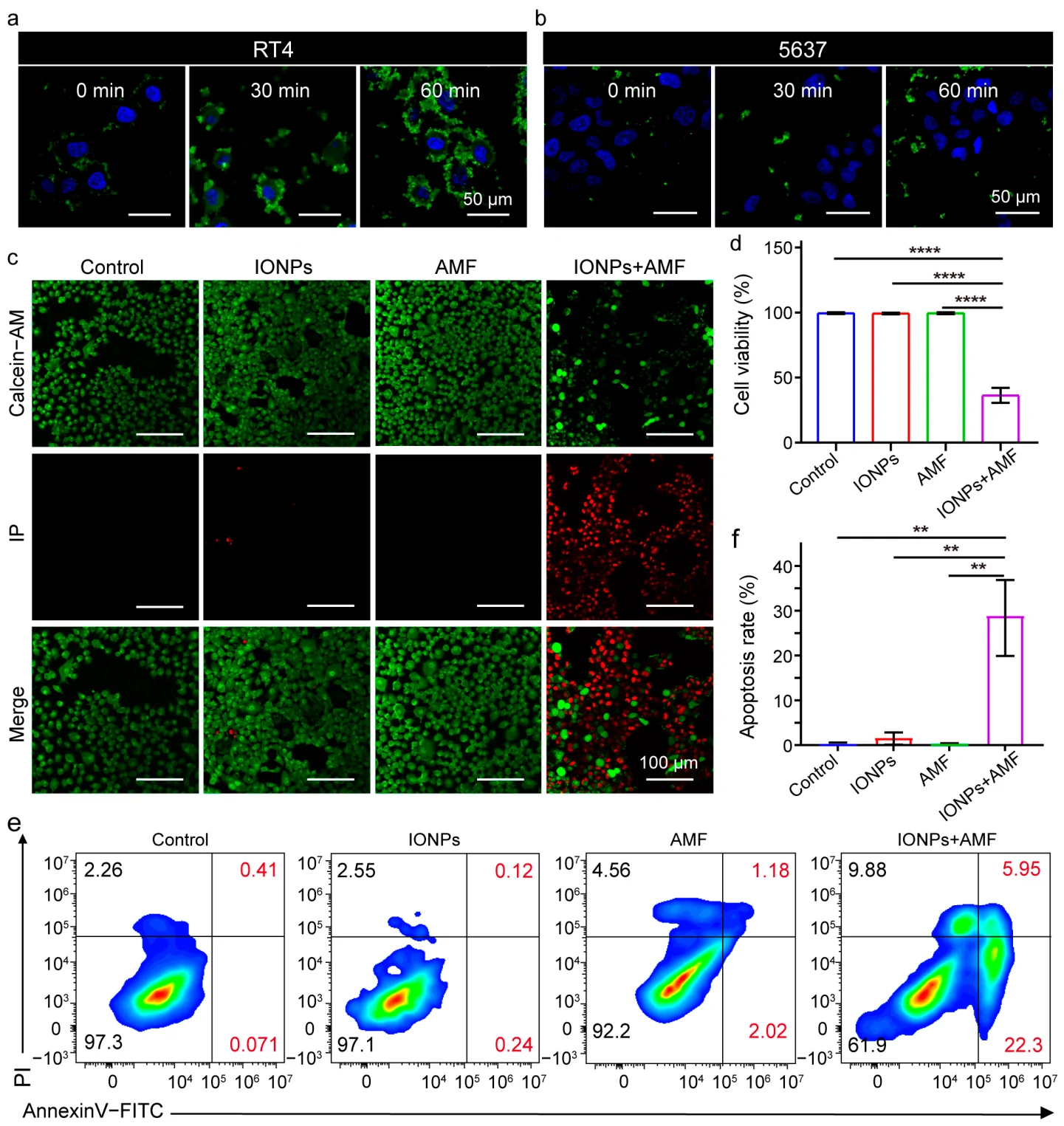
In vitro MHT effect. CLSM images of (**a**) RT4 and (**b**) 5637 cells after incubation with anti-FGFR3-IONPs for 0, 30, and 60 min. Nuclei and IONPs were labeled with Hoechst 33342 (blue) and FITC (green), respectively. Scale bar: 50 μm. (**c**) CLSM images of RT4 cells after MHT. Live cells and dead cells were stained with calcein-AM (green) and PI (red), respectively. Scale bar: 100 μm. (**d**) Quantitative analysis of cell viability across treatment groups. (**e**) Flow cytometric determination of apoptosis and (**f**) corresponding quantitative apoptosis rate in RT4 cells treated with PBS (control), IONPs, AMF, and IONPs+AMF. Data are displayed as mean ± SD (*n* = 3). ** *p* < 0.01; **** *p* < 0.0001.

The expression levels of FGFR3 in RT4 cells were subsequently analyzed using quantitative PCR. A significant increase in FGFR3 mRNA expression was found in RT4 cells compared to 5637 cells (Figure S5). Furthermore, bioinformatic analysis revealed that FGFR3 transcript levels were significantly higher in papillary bladder tumors compared to normal tissues (*p* < 0.001), whereas no significant difference was observed in non-papillary subtypes (Figure S6). These data showed the potential of FGFR3 as a specific target. To evaluate the selectivity of anti-FGFR3-IONPs to target FGFR3, the anti-FGFR3-IONPs were labeled with FITC (green) to prepare IONP-FGFR3-FITC, which was co-incubated with RT4 cells and 5637 cells, respectively. As shown in CLSM images in Figure 3a,b, a strong green fluorescence signal around the RT4 cell membrane could be observed after 60 min of incubation. In contrast, green fluorescence intensity around the 5637 cell was weaker after the same incubation. This suggested that the coating of anti-FGFR3 antibodies enables IONPs to efficiently target FGFR3 on the membrane of RT4 cells, which is beneficial for subsequent tumor-targeting MHT mediated by anti-FGFR3-IONPs.

In order to assess whether IONP+AMF treatment could provide an antitumor effect, the cytotoxic effects and their impacts on the proliferative capacity of tumor cells were studied. After IONP+AMF treatment, AM/PI staining was conducted to assess the cytotoxicity. As displayed in Figure 3c, RT4 cells in the control, IONP, and AMF groups showed predominantly green fluorescence (live cells) after co-cultivation. In contrast, cells in the IONP+AMF group exhibited extensive red fluorescence, indicating that the IONP+AMF treatment resulted in a marked elevation of the tumor cell death rate, with cell viability decreased to 36.33% (Figure 3d). In addition, the IONP+AMF treatment exhibited significantly enhanced in vitro antitumor effect compared with the non-targeted magnetothermal treatment (Figure S7). Flow cytometry experiments were further conducted to assess the antitumor efficiency of IONP+AMF treatment. As presented in Figure 3e,f, the IONP+AMF group induced an apoptosis rate of 28.25%, which substantially surpasses the corresponding values from the control, IONP, and AMF groups, respectively. These results demonstrated the high efficacy of in vitro MHT mediated by the anti-FGFR3-IONPs, providing a foundational basis for developing a precision in vivo MHT.

### 2.3. IONPs Induced Augmented Apoptosis in BCa Cells

**Figure 4:**
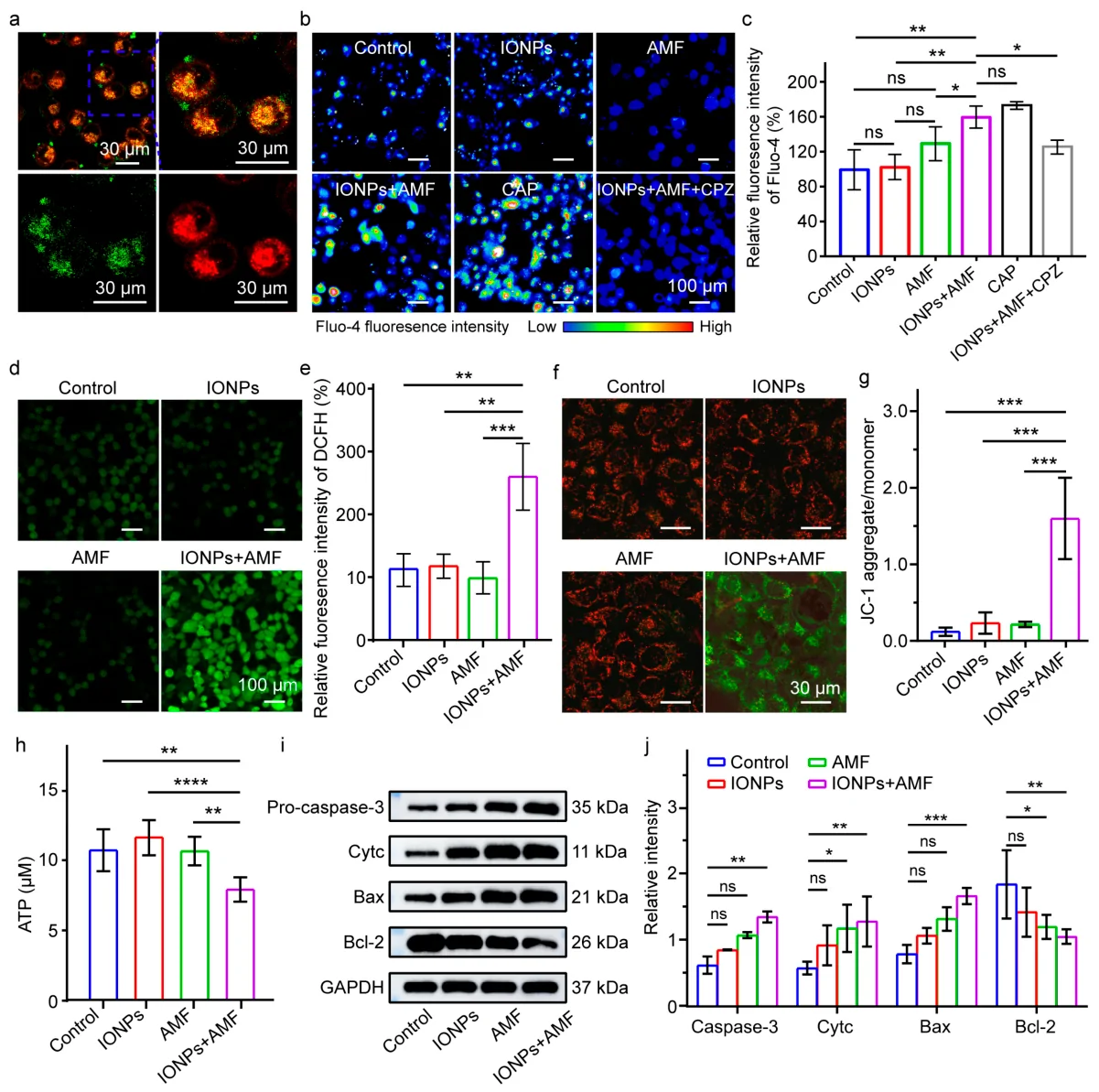
Mechanism of MHT-induced RT4 cell death. (**a**) CLSM images of co-localization of anti-FGFR3-IONPs (green) and ER (red). (**b**) Representative color maps from CLSM images of RT4 cells loaded with Ca^2+^ indicator Fluo-4 (green) under different treatments (control, IONPs, AMF, IONPs+AMF, CAP, IONPs+AMF+CPZ), and (**c**) quantitative results of relative fluorescence intensity of Fluo-4. (**d**) Representative CLSM images of RT4 cells stained with a ROS indicator (DCFH-DA, green) and (**e**) quantification of DCFH-DA relative fluorescence intensity. (**f**) CLSM images of JC-1-stained RT4 cells and (**g**) the quantitative ratio of red fluorescent aggregates (polarized mitochondria, normal ΔΨm) to green fluorescent monomers (depolarized mitochondria, decreased ΔΨm). (**h**) Quantification of intracellular ATP levels (μM) in RT4 cells. (**i**) Western blot analysis of protein expression (Pro-caspase-3, Cytc, Bax, and Bcl-2) in RT4 cells after treatments and (**j**) corresponding densitometric quantification. Data are displayed as mean ± SD (n = 3). ns, not significant; * *p* < 0.05; ** *p* < 0.01; *** *p* < 0.001; **** *p* < 0.0001.

The encouraging in vitro antitumor effect of MHT motivated us to delve deeper into the mechanisms of augmented apoptosis. The TRPV1 expression was analyzed by real-time quantitative PCR. As shown in Figure S8, TRPV1 was endogenously overexpressed on the endoplasmic reticulum (ER) in RT4 cells, compared with 5637 cells. We next performed CLSM imaging experiments to examine the uptake and intracellular localization of anti-FGFR3-IONPs, which were labeled with FITC. As presented in Figure 4a, after coincubation with IONP-FGFR3-FITC for 1 h, the co-localization of FITC and red ER-tracker fluorescence was clearly observed, providing a basis for precise magnetothermal activation of ER-TRPV1.

The response of ER-TRPV1 to the magnetothermal effect of IONPs was investigated by monitoring intracellular Ca^2+^ dynamics in RT4 cells. Before the different treatments, the RT4 cells were loaded with a fluorescent Ca^2+^ indicator (Fluo-4). Figure 4b shows the representative color maps from CLSM images of RT4 cells loaded with Fluo-4 (green). Figure 4c shows the corresponding quantification of Fluo-4 relative fluorescence intensity in each group. In the IONP+AMF group, RT4 cells exhibited a remarkable increase in intracellular Ca^2+^ fluorescence intensity in comparison with the control, IONP, and AMF groups. No significant difference in relative fluorescence intensity of Fluo-4 was observed between the control and IONP or between the control and AMF groups, indicating that neither IONPs nor AMF alone triggers detectable intracellular Ca^2+^ elevation. The relative Fluo-4 fluorescence intensity in the IONP+AMF group was significantly higher than that in the control (*p* = 0.0022), IONP (*p* = 0.0011), and AMF (*p* = 0.0213) treatment groups. No statistical difference was found between IONPs+AMF and the canonical Ca^2+^ agonist CAP group. Importantly, when RT4 cells were pretreated with the TRPV1 selective antagonist capsazepine (CPZ), the relative fluorescence intensity of Fluo-4 was substantially attenuated in the IONP+AMF+CPZ group. The CPZ co-treatment group showed a significant difference from the IONP+AMF group (*p* = 0.0386). These findings collectively demonstrated that the magnetothermal effect of IONPs generated under AMF effectively activates ER-TRPV1, leading to Ca^2+^ release in RT4 cells.

To further investigate cellular stress responses downstream of Ca^2+^ signaling in the IONP+AMF group, the DCFH-DA probe was loaded into cells to monitor intracellular ROS levels. Specifically, IONPs with peroxidase-like activity under AMF catalyzed the reaction of the non-fluorescent DCFH-DA to DCFH, which was then oxidized by ROS to generate fluorescent DCF, with the fluorescence intensity directly reflecting intracellular ROS levels. CLSM images revealed a significant increase in ROS in the IONP+AMF group compared with other groups (Figure 4d,e). This explosive ROS production promoted cell death. Specifically, high ROS levels inhibited the activity of the plasma membrane Ca^2+^ ATPase (PMCA), thereby hindering intracellular Ca^2+^ efflux. This further exacerbated Ca^2+^ accumulation, ultimately triggering Ca^2+^ overload-associated cell death. ROS accumulation directly attacked mitochondrial membrane structures, inducing sustained opening of the mitochondrial permeability transition pore. To further examine this, the membrane potential (ΔΨm) of mitochondria was assessed by JC-1 staining. JC-1 can act as a marker of mitochondrial membrane potential. The red-emitting aggregates form in healthy cells (high membrane potential), while green-emitting monomers accumulate in damaged cells (low membrane potential), and thus a decreased red/green ratio indicates mitochondrial depolarization and early apoptosis. In the IONP+AMF group, the ratio between red fluorescent aggregates and green fluorescent monomers increased approximately 13-fold within 24 h compared to the control group, indicating significant mitochondrial depolarization (Figure 4f,g). The loss of ΔΨm suggests impairment of the mitochondrial electron transport chain, which can further amplify ROS production and promote the release of pro-apoptotic factors into the cytoplasm. Consistently with this observation, intracellular ATP levels were markedly reduced in the IONP+AMF group versus the other groups (Figure 4h).

To dissect the molecular mechanisms of apoptosis augmentation in the IONP+AMF group, we assessed the mRNA transcript levels of Bax, Bcl-2, Pro-caspase-3, and Cytochrome c (Cytc) via qRT-PCR. The IONP+AMF treatment resulted in significantly upregulated expression of pro-apoptotic genes (Bax, Pro-caspase-3, and Cytc) and downregulated the anti-apoptotic Bcl-2 expression in RT4 cells, relative to the control, IONP, and AMF groups (Figure S9). The transcriptional changes were consistent with alterations observed at the protein level in Western blot analysis (Figure 4i,j and Figure S10). Taken together, these findings indicate that the IONP+AMF treatment exerts its antitumor activity through the mitochondrial-dependent apoptosis pathway.

### 2.4. RNA-Sequencing Analysis

**Figure 5:**
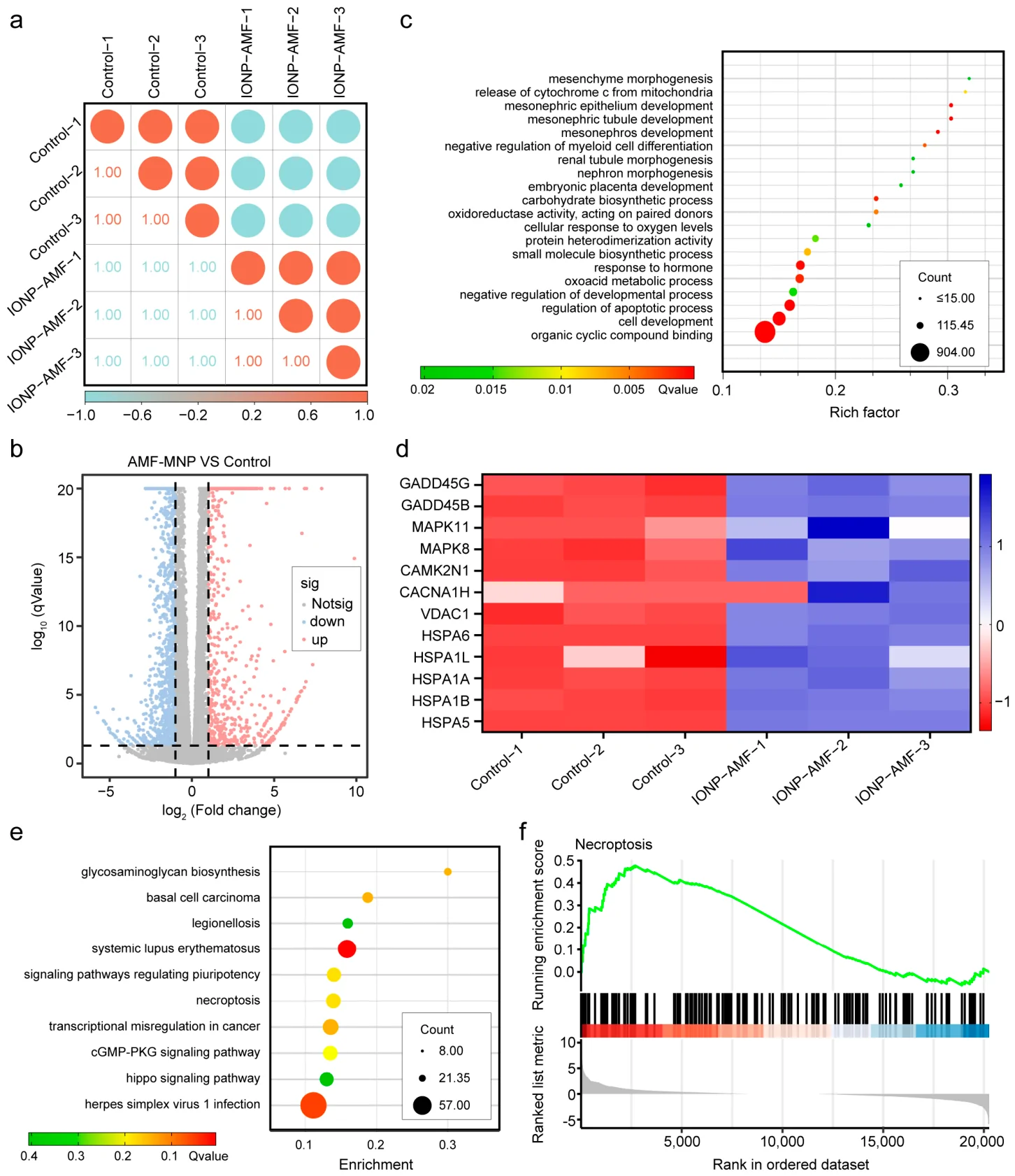
RNA-seq analysis of RT4 cells following IONP+AMF treatment for 24 h. (**a**) Correlation coefficient matrix illustrating reproducibility among RNA-seq samples. (**b**) Volcano plot depicting upregulated and downregulated genes in the IONP+AMF group. (**c**) GO enrichment analysis of DEGs. (**d**) Heatmap showing the expression profiles of DEGs in control and IONP+AMF groups. (**e**) Kyoto Encyclopedia of Genes and Genomes (KEGG) pathway enrichment analysis of DEGs. (**f**) Gene set enrichment analysis (GSEA) reveals positive enrichment of apoptosis-related gene sets following IONP+AMF treatment.

In addition to causing mitochondrial dysfunction, intracellular Ca^2+^ overload can also affect numerous biological processes in cancer cells that are closely related to malignant phenotypes. To confirm this, we performed RNA-sequencing on RT4 cells to exploit the global transcriptome alterations following IONP+AMF treatment. The gene expression profiles demonstrated high consistency between technical replicates (all pairwise correlation coefficients > 0.95), confirming the reproducibility of the experimental system and the reliability of the subsequent data analysis (Figure 5a). Compared with control group, the IONP+AMF group induced widespread transcriptional reprogramming, revealing 2638 significantly different expressed genes (DEGs) (q < 0.05, |log_2_FC| > 1), comprising 1206 upregulated and 1432 downregulated genes (Figure 5b). Gene ontology (GO) enrichment analysis revealed that IONP+AMF treatment significantly modulated several core biological processes. The most prominently enriched terms were associated with mesenchymal morphogenesis, carbohydrate biosynthesis processes, negative regulation of developmental processes, and the fine-tuning of apoptosis pathways (Figure 5c). Notably, we observed coordinated downregulation of key regulators within apoptosis-related modules, including MAPK11 and MAPK8 of the MAPK signaling pathway, calcium (Ca^2+^) homeostasis regulators (CAMK2N1, CACNA1H, VDAC1), and multiple genes encoding heat shock proteins (HSPA1L, HSPA1A, HSPA1B, HSPA5, HSPA6) (Figure 5d). These findings suggest that IONP+AMF treatment disrupts Ca^2+^ signaling and protein homeostasis, thereby affecting cellular processes such as cell proliferation.

Complementing the above-mentioned observations, KEGG pathway analysis revealed significant enrichment of DEGs in pathways closely linked to tumor progression and cell death, including the HIF-1, p53, and MAPK signaling pathways, as well as necrotic apoptosis (Figure 5e). Global expression trends visualized by GSEA further confirmed that necrosis-related gene sets were significantly suppressed following IONP+AMF treatment (Figure 5f). Collectively, the transcriptomic data provide a systematic view of how IONP+AMF treatment orchestrates key signaling networks, leading to dysregulation of Ca^2+^ homeostasis and HSP expression. This coordinated modulation ultimately suppresses necroptosis while promoting apoptotic cell death, offering a mechanistic rationale for the antitumor effects of IONP+AMF treatment.

### 2.5. Therapeutic Efficacy of Intravesical MHT In Vivo

Given the encouraging in vitro results, we further evaluated the efficacy of FGFR3-targeted MHT in an orthotopic RT4 BCa model. Specifically, the orthotopic BCa mice were established by implantation of luciferase-labeled RT4 cells. This cell line, established from a primary tumor, maintains strong epithelial characteristics, limited invasive capacity, and a structured growth pattern under in vivo conditions [38,41]. Successful tumor engraftment was confirmed on day 14 post-inoculation. As shown in Figure S11a, the bioluminescence images of the bladder obtained using an in vivo imaging system (IVIS) showed a strong bioluminescent signal compared to that acquired from normal mice. Figure S11b shows the MR images of the bladder areas in mice. For BCa mice, a significant negative contrast (T2 signal) in the bladder region was observed in the images, suggesting that the irregular tumor tissues grew along the bladder wall. Figure S11c displays the H&E-stained sections of bladder tissues acquired from normal and BCa mice. In the BCa mice group, the dense nests of tumor cells almost filled the bladder lumen, and the tumor tissue exhibited hyperchromatic nuclei, increased nuclear-to-cytoplasmic ratio, and papillary architecture with fibrovascular cores, consistent with the well-differentiated, low-grade nature of this cell line. In sharp contrast, the normal mice group displayed an intact transitional epithelium with orderly stratified architecture and no evidence of tumor cell colonization. The RT4 xenograft model was a grade I papillary bladder urothelial carcinoma and luminal-type non-muscle-invasive bladder cancer (NMIBC). It offers a physiologically stable and clinically meaningful platform for assessing early-stage bladder cancer interventions. Collectively, these results demonstrate that orthotopic BCa mice were successfully established for the evaluation of the antitumor effect of FGFR3-targeting MHT in vivo.

**Figure 6:**
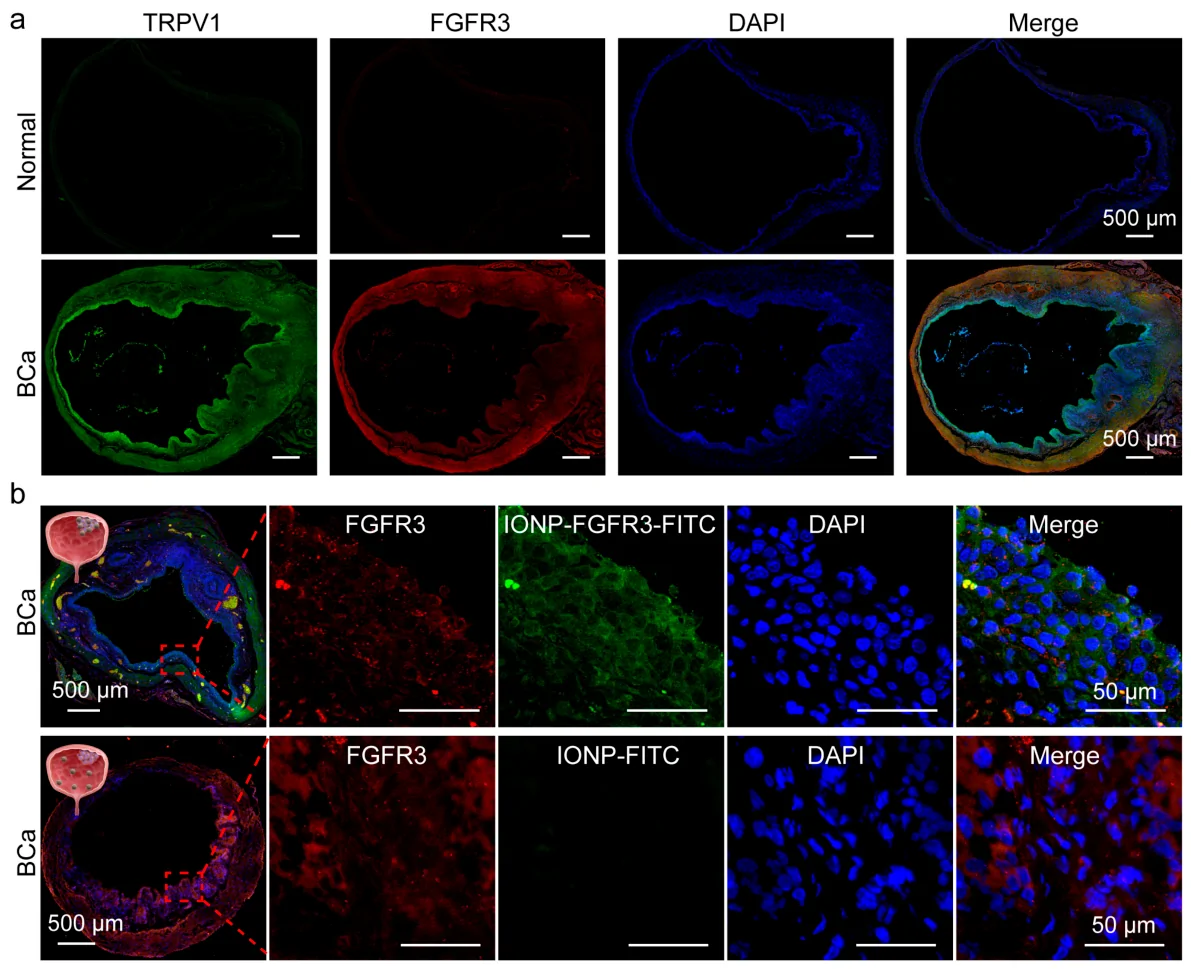
Co-expression of FGFR3 and TRPV1 in BCa tissues and FGFR3-targeting of IONPs. (**a**) Immunofluorescence staining of FGFR3 (red) and TRPV1 (green) in bladder tissues from normal and BCa mice. (**b**) Immunofluorescence staining of FGFR3-targeting IONPs functionalized with FITC-labeled anti-FGFR3 antibodies (IONP-FGFR3-FITC, green) and FITC-labeled IONPs (IONP-FITC) without antibody functionalization.

To further study the FGFR3-targeting effect of IONPs in vivo, we first isolated the bladder tissues from normal and BCa mice, and visualized the specific co-expression of FGFR3 and TRPV1 in these tissues by immunofluorescence staining. As shown in Figure 6a, the BCa tissues exhibited much higher fluorescence intensity of FGFR3 and TRPV1 than that in normal mice. The results validated that both FGFR3 and TRPV1 were highly expressed in BCa tissue in comparison with normal bladder tissue. Based on this, FGFR3-targeting IONPs were prepared by functionalization of IONPs with FITC-labeled anti-FGFR3 monoclonal antibodies (IONP-FGFR3-FITC). The FGFR3-targeting effect of IONPs was then examined. The IONPs and FGFR3-targeted IONPs were prepared by functionalization of IONPs with FITC. After intravesical instillation, the IONP-FGFR3-FITC selectively targeted the FGFR3-expressing BCa cells through antigen–antibody binding (Figure 6b). In contrast, the IONPs without antibody coatings (IONP-FITC) failed to target the FGFR3-expressing cells, showing a considerably decreased green fluorescence signal targeting efficacy in BCa tissues. These results suggest that an anti-FGFR3 antibody can effectively improve the tumor-targeting efficacy of IONPs.

**Figure 7:**
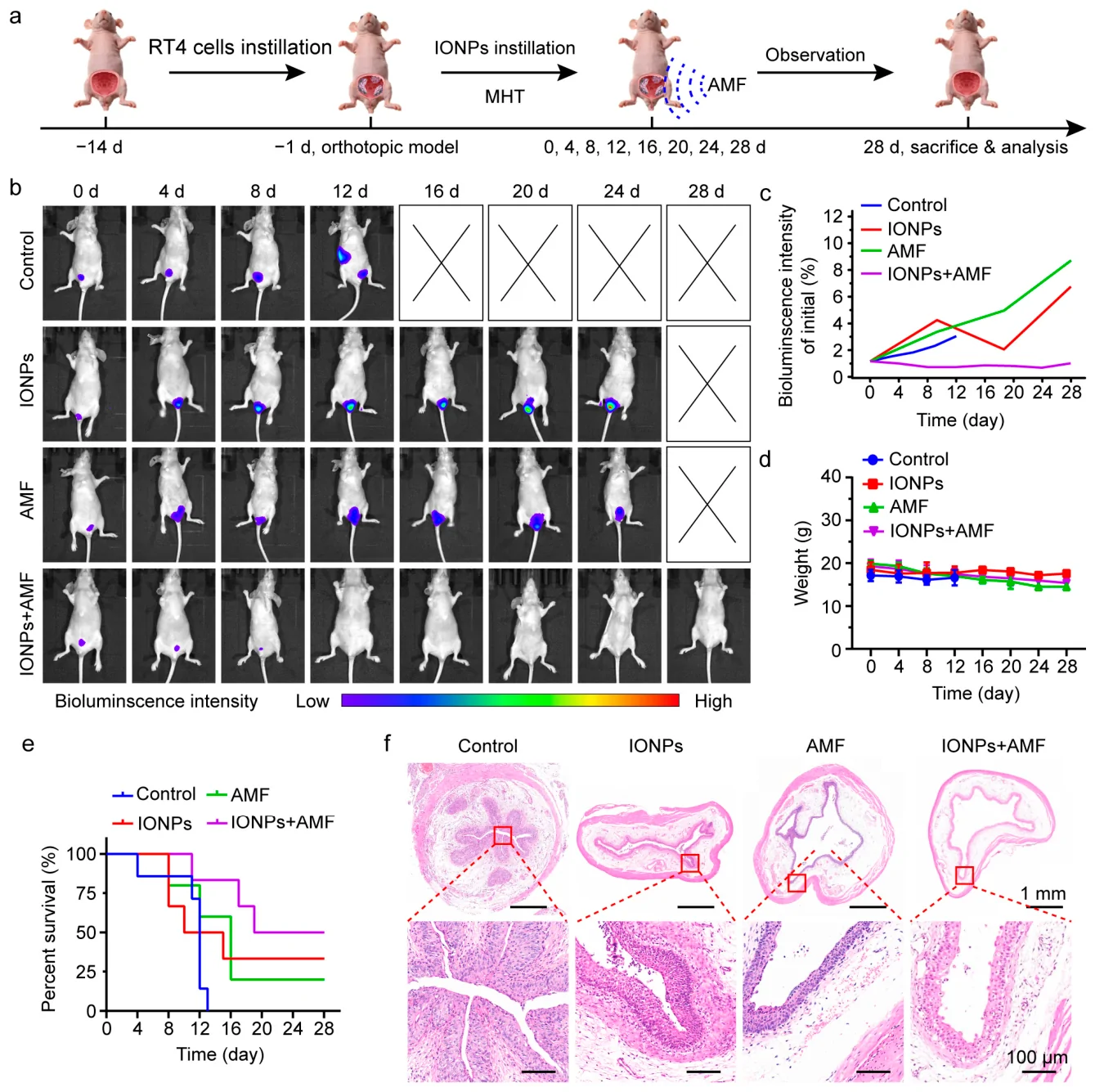
Evaluation of the tumor inhibition effect of intravesical targeted MHT in vivo. (**a**) Illustration of the treatment regimen for orthotopic BCa-bearing BALB/c nude mice. (**b**) Longitudinal bioluminescence imaging of BCa 0, 4, 8, 12, 16, 20, 24, and 28 days. (**c**) Bioluminscence intensity of initial (%) of the BCa tissue in different groups (control, IONPs, AMF, IONPs+AMF). (**d**) post-treatments in different groups (control, IONPs, AMF, IONPs+AMF) and tumor bioluminescence quantification (n = 5). (**e**) Body weight changes and survival curves of mice in different groups during the treatment periods (n = 5). (**f**) Representative images of H&E staining of BCa sections from mice in different groups. Data are displayed as mean ± SD (n = 3).

We further exploited the in vivo antitumor effect of our FGFR3-targeted MHT approach by intravesical instillation of IONPs in orthotopic RT4 BCa mice. We randomly classified the BCa mice into four groups, including the control, IONP, AMF, and IONP+AMF groups. Figure 7a presents a schematic illustration of the treatment schedule. In the IONP+AMF group, in vivo thermometry showed that the temperature of the bladder in mice increased to over 42 °C after intravesical instillation of anti-FGFR3-IONPs under AMF exposure (Figure S12). After that, tumor progression in different groups was monitored through longitudinal monitoring via bioluminescence imaging. As displayed in Figure 7b,c, the control group exhibited rapid tumor growth, with evidence of metastasis observed on the 12th day and mortality within 16 days. In contrast, the IONP+AMF group showed obvious tumor inhibition, with some mice showing no detectable fluorescence signal of the tumor at the end of treatment. The IONP+AMF treatment yielded a substantially higher tumor inhibition rate compared to the control, IONP, and AMF groups. The weight of the mice’s bodies did not show any significant decrease during the entire treatment period (Figure 7d). In addition, the mice in the IONP+AMF group showed a significantly improved survival rate compared to other groups (Figure 7e), suggesting an effective antitumor effect associated with this treatment.

The superior antitumor effect of the IONP+AMF treatment was further validated by harvesting the tumor at the experimental endpoint from the different groups for histological and immunohistochemical analysis. As shown in H&E staining results, the IONP+AMF group exhibited the most effective therapeutic impact on tumors, with no obvious tumor lesions (Figure 7f). In comparison with the IONP+AMF group, the control, IONP, and AMF groups showed an obvious thickened bladder wall due to extensive tumor cell infiltration. Ki-67 staining results further demonstrated that the IONP+AMF group showed a robust capacity to inhibit tumor growth, indicating significantly greater antiproliferative efficacy compared to the others (Figure S13). Furthermore, the bladder tissues were also visualized using TUNEL staining. A significant increase in DNA damage in tumor cells following IONP+AMF treatment was also found in the TUNEL staining images, indicating a substantial acceleration of the apoptotic process relative to the control, IONP, and AMF groups (Figure S14). Taken together, our results provide substantial evidence that the intravesical FGFR3-targeted MHT strategy effectively suppresses tumor growth and exhibits minimal harm to normal tissues, eventually leading to improved survival rates.

## 3. Discussion and Conclusions

The FGFR3-targeting strategy has attracted considerable attention in BCa treatment recently. This is because the transmembrane FGFR3 leads to the development of aggressive tumors by upregulating cell proliferation and migration in BCa [34,42,43,44,45,46,47,48]. The RT4 cell line, owing to high FGFR3 expression, was chosen as the FGFR3-positive experimental model compared with non-malignant urothelial cell lines (such as SV-HUC-1 cells) [49]. RT4 represents a well-differentiated, low-grade luminal non-muscle-invasive bladder cancer (NMIBC) model that harbors the FGFR3-TACC3 fusion gene. As such, it is highly relevant for FGFR3-driven BCa, which constitutes a major subset of NMIBC (~80% of cases carry FGFR3 alterations). Thus, FGFR3 is regarded as an important target [42,45]. The 5637 cell line with low FGFR3 expression was selected as the control, thereby allowing us to verify that the observed selective targeting effects are specifically mediated by FGFR3 recognition, rather than non-specific cellular adsorption. However, it is still unknown whether the anti-FGFR3 antibody-conjugated IONPs after intravesical instillation can target tumors, enhance nanodrug accumulation, and improve therapeutic efficacy.

We first demonstrated that the FGFR3-targeting IONPs after intravesical instillation can specifically bind to FGFR3 on RT4 cells, without obvious adhesion to the target site and systemic circulation of nanoparticles, because of the continuous addition of fresh urine [13]. The anti-FGFR3-IONPs under AMF exposure produced a robust antitumor effect. It showed significantly upregulated pro-apoptotic gene expression of Bax, Pro-caspase-3, and Cytc in RT4 cells, and downregulated the anti-apoptotic gene Bcl-2. Notably, TRPV1 is a non-selective cation channel, which can be activated by thermal stimuli (>43 °C), including tumor cell apoptosis [37,50,51]. In our work, the excellent cellular uptake of IONPs was a prerequisite for ER-TRPV1 magnetothermal activation, which subsequently increased the local temperature above 42 °C and caused cytosolic Ca^2+^ accumulation and overloading, as reported previously [29,39,52,53]. However, this RT4 model may not fully represent more aggressive, FGFR3-independent subtypes such as T24 or UM-UC-3. Therefore, our FGFR3-targeted strategy is most suitable for FGFR3-driven luminal tumors, while FGFR3-independent aggressive subtypes would require alternative targeting approaches.

The antitumor effect of our MHT strategy is not exclusively attributable to TRPV1-mediated calcium overload, but rather results from a combination of specific and non-specific thermal effects. Hyperthermia is known to induce a broad spectrum of cellular responses that collectively contribute to tumor cell death. First, we demonstrated that TRPV1-mediated Ca^2+^ overload plays a critical role in initiating mitochondrial apoptosis and decreasing cellular viability. Second, as reported in previous studies, MHT can induce heat stress responses mediated by upregulating heat shock proteins (HSPs), such as HSP70 and HSP90, which can decrease the efficacy of localized heat treatment [54]. In addition, MHT also caused non-specific thermal effects, including protein denaturation and direct cell membrane damage. We acknowledge that this antitumor mechanism from a combination of specific and non-specific thermal effects deserves thorough investigation and elucidation in future studies.

Nevertheless, our MHT monotherapy cannot completely eradicate tumors. This limitation can be attributed to three main factors: (i) heterogeneous heat distribution within the tumor bulk during AMF application; (ii) the possible upregulation of cytoprotective heat shock proteins in the surviving cell population; and (iii) the inherent resistance conferred by the hypoxic and acidic tumor microenvironment against oxidative damage. The inability of the monotherapy to completely eradicate the tumor leaves a risk of recurrence. To counteract the high recurrence rate of BCa, immunotherapeutic intervention stands out as the most viable adjunctive modality. The core rationale of cancer immunotherapy lies in reactivating the host immune system to recognize and eliminate tumor cells. Therefore, rationally designed combination regimens, particularly those integrating our MHT with immune checkpoint inhibitors, which exploit hyperthermia-induced immunogenic cell death to prime systemic antitumor immunity, are strongly warranted for future translational exploration [32,45].

Multi-dimensional safety evaluation is essential for our anti-FGFR3-IONP intravesical therapy. In previous work, IONPs are already approved as MRI contrast agents and iron supplements by the U.S. Food and Drug Administration (FDA) and China’s National Medical Products Administration (NMPA) [28,55]. Moreover, IONP-based MHT is FDA-approved for glioma and prostatic cancer treatment [56]. In our study, H&E staining results showed no discernible pathological damage in major organs in mice (Figure S15), thus ensuring that sufficient nanoparticles accumulate at the tumor site without systemic circulation and greatly reducing biosafety concerns [32]. Notably, our safety data presented are relatively limited. We plan to conduct multi-dimensional safety evaluation in our future work. This will include: (i) local bladder toxicity assessment via H&E and Masson’s trichrome staining at multiple post-treatment time points; (ii) systemic biodistribution analysis by measuring iron content in major organs using ICP-MS; (iii) serum biochemistry profiling to evaluate hepatic and renal function; and (iv) long-term observation of bladder fibrosis and tissue remodeling. This will position intravesical instillation MHT as a highly translatable therapeutic strategy.

In summary, we developed novel FGFR3-targeting IONPs with good biocompatibility and demonstrated an efficient intravesical MHT strategy for BCa. Following intravesical instillation, these IONPs efficiently targeted FGFR3 on RT4 cells. The magnetothermal effect of IONPs enhanced the ROS-generating ability and activated ER-TRPV1 in RT4 cells, which caused severe mitochondrial dysfunction and ultimately induced augmented apoptosis. The application of intravesical FGFR3-targeting MHT successfully resulted in greater suppression of tumor growth and improved survival rate of mice, compared to the control, IONP, and AMF-treated groups. Together, the ER-TRPV1-specific IONP-based MHT strategy holds significant potential as a transformative modality toward BCa therapeutic application.

## 4. Materials and Methods

### 4.1. Materials

The reagents FeO(OH) and 3,4-dihydroxyhydrocinnamic acid (DHCA) were bought from Macklin (Shanghai, China). The oleic acid was obtained from Sigma-Aldrich (Shanghai, China). The 1-ethyl-3-(3-dimethylaminopropyl) carbodiimide (EDC), N-hydroxysuccinimide (NHS), capsaicin, and capsazepine (CPZ) were sourced from Aladdin (Shanghai, China). Anti-FGFR3 antibody was purchased from Proteintech (Wuhan, China). The Fluo-4 probe was obtained from Invitrogen (Shanghai, China).

### 4.2. Preparation of Fe_3_O_4_ Nanoparticles

Monodisperse IONPs (Fe_3_O_4_) with a diameter of ~20 nm were synthesized via a high-temperature thermal decomposition method. Briefly, a mixture of 0.18 g (2 mmol/L) FeO(OH) and 3.39 g (6 mmol/L) oleic acid (OA) in 5.00 g squalane was heated and kept at 180 °C for 2 h under nitrogen flow. The resulting mixture was then rapidly heated to 320 °C and refluxed for an additional 1 h. The IONPs coated with OA were centrifuged by adding a mixed solvent of anhydrous ethanol and hexane for 3 times.

To render the nanoparticles water-dispersible, the OA ligands on the surface of IONPs were replaced with DHCA via a ligand exchange process. Specifically, 30 mg of IONPs and 70 mg of DHCA were dispersed in tetrahydrofuran (THF, 20 mL). The THF dispersion was maintained at 55 °C and stirred for 4 h. After that, the mixture was centrifuged, and the IONP-DHCA was obtained and dispersed in deionized (DI) water.

### 4.3. Preparation of Anti-FGFR3 Antibody-Coated Fe_3_O_4_ Nanoparticles

The functionalization of anti-FGFR3 antibodies onto IONP-DHCA was carried out via an EDC/NHS coupling reaction. Briefly, 10 mg of IONP-DHCA was mixed with EDC (19.2 mg) and incubated for 30 min. The IONPs were then obtained after centrifugation and were resuspended in PBS (pH 7.4). A total of 3 mg of anti-FGFR3 antibodies and 22.6 mg of NHS were reacted with IONPs in a PBS suspension for the coupling reaction for 3 h on a shaker. Finally, the IONP-FGFR3 was collected after centrifugation and resuspended in PBS.

### 4.4. Cell Toxicity Assay

The human BCa cell line 5637 and the RT4 cell line were obtained from Yuchi (Shanghai, China) Biotechnology Co., Ltd. The cytotoxicity of the IONPs was measured in triplicate via a CCK-8 assay. Briefly, cells seeded in 96-well plates were incubated with IONPs at different Fe concentrations (0, 20, 40, 60, 80, 100 μg/mL) for 24 h. After that, a CCK-8 assay was performed by recording the absorbance at 450 nm using a multi-mode microplate reader to evaluate the cytotoxicity of the IONPs.

### 4.5. Evaluation of Antitumor Efficacy of MHT In Vitro

RT4 cells were divided into 4 groups, including the control, IONP, AMF, and IONP+AMF groups. During IONP+AMF treatment, the RT4 cells were incubated with IONP-FGFR3 (80 μg/mL) and exposed to AMF (315 kHz, 300 Oe). Live and dead cells were stained with calcein-AM and propidium iodide (PI), respectively. Imaging was conducted by a confocal laser scanning microscope (CLSM, Olympus FV3000, OptoSigma, Les Ulis, France), and qualitative analysis of the obtained images was further assessed to evaluate cell viability. Each experiment was independently repeated 3 times.

Apoptosis in RT4 cells was further evaluated by flow cytometry analysis. Briefly, cells were incubated with medium containing IONP-FGFR3 (Fe: 80 μg/mL) for 20 min. After that, the cells were collected and washed twice with cold PBS, and stained with Annexin V-FITC and PI. Fluorescence was subsequently analyzed by a flow cytometer.

### 4.6. Measurement of Ca^2+^ Influx and Intracellular Ca^2+^ Generation

RT4 cells were loaded with a Ca^2+^ indicator (Fluo-4) and incubated at 37 °C for 30 min. After staining, the Fluo-4-containing solution was replaced with IONP-FGFR3-containing medium (Fe: 80 μg/mL) for an additional incubation (20 min). The dishes were washed with PBS, and fluorescent changes in Fluo-4 in the cells were observed by CLSM.

### 4.7. Detection of Intracellular ROS Production

Intracellular ROS levels in cells were assayed by the DCFH-DA fluorescent probe. RT4 cells were cultured in 35 mm confocal dishes for 24 h. The medium was then replaced with medium containing IONP-FGFR3 (Fe: 80 μg/mL). After AMF exposure, the cells were stained with DCFH-DA, and the fluorescence intensity changes in DCFH-DA were visualized using CLSM.

### 4.8. Detection of Mitochondrial Membrane Potential

RT4 cells were cultured in medium containing IONP-FGFR3 (Fe: 80 μg/mL) in 35 mm confocal dishes. After AMF exposure, cells were collected and lysed using the kit-provided lysis buffer to release ATP. The lysates were reacted with a luciferin–luciferase mixture, and luminescence intensity, which is proportional to ATP concentration, was measured using a chemiluminometer. The mitochondrial membrane potential (ΔΨm) changes in RT4 cells were determined by staining cells with the JC-1 fluorescent probe and imaged by CLSM.

### 4.9. Western Blot

RT4 cells in different groups were washed with ice-cold PBS and lysed in RIPA buffer supplemented with PMSF. Total protein concentration was assayed via a BCA method. Equal protein aliquots were separated by SDS-PAGE, transferred to PVDF, blocked with 5% non-fat milk/TBST for 3 h, and probed overnight at 4 °C with primary antibodies targeting Bcl-2, BAX, Pro-caspase-3, and Cytc. Membranes were washed with TBST, incubated with horseradish peroxidase-conjugated secondary antibodies for 1 h, and visualized via a gel imaging system.

### 4.10. Gene Expression Analysis

Total RNA was extracted from RT4 cells in different groups using TRIzol reagent. Complementary DNA (cDNA) was fabricated from the extracted RNA. qPCR was subsequently conducted to evaluate the gene expression levels. All reactions were run under standard cycling conditions with an annealing temperature of 60 °C. According to the ΔΔCt method, relative gene expression was calculated and normalized to the control group. All primers used in the experiments are listed in Table S1 and were synthesized by Service Bio (Wuhan, China).

### 4.11. RNA-Sequencing Analysis

Total RNA was extracted using Total RNA Extractor (Trizol) reagent, and RNA purity and concentration were measured by a NanoDrop ND-2000 spectrophotometer (Thermo Fisher Scientific, Waltham, MA, USA). Sequencing was performed on the DNBSEQ-T7 platform. Differential expression analysis comparing the “IONP+AMF” with the control group was performed via the DESeq2 package (version 1.53.2). Functional enrichment analysis, including GO and KEGG pathway analyses, was conducted using the clusterProfiler (version 4.20.0) and topGO software packages (version 2.36.0), respectively.

### 4.12. Antitumor Efficacy of Intravesical MHT In Vivo

All animal experiments and procedures were approved by the Animal Ethics Committee of Fourth Military Medical University and performed in strict accordance with institutional guidelines. The study also adhered to the ARRIVE guidelines. BALB/c nude mice (weighing 17 ± 1 g, female) were obtained from Chongqing Tengxin Biotechnology Co., Ltd. (Chongqing, China).

An orthotopic BCa model was established via intravesical inoculation of RT4 cells. Briefly, a 24-gauge intravenous indwelling needle cannula, lubricated with glycerol, was gently inserted into the bladder through the urethra. After urine removal, the bladder was washed three times with 100 μL PBS. A 50 μL suspension of RT4 cells (4 × 10^7^ cells/mL in Matrigel) was then injected into the bladder. The urethral orifice was gently clamped with forceps to prevent tumor cells from leaking. After 1 h, the ligation was released, and the mice were transferred to a clean cage for recovery.

Orthotopic BCa mice were randomly divided into four groups (n = 5) for an in vivo therapeutic study. The control group received intravesical instillation of PBS, while the IONP group received instillation of IONP-FGFR3 (2.5 mg/kg). The AMF group was subjected to AMF exposure for 10 min. The IONP+AMF group received intravesical instillation of IONP-FGFR3 followed by AMF exposure for 10 min (315 kHz, 300 Oe). AMF exposure was conducted once every 4 days for a total of 7 cycles. During the treatment period, tumor progression was recorded every 4 days using bioluminescent imaging (IVIS Spectrum) following intraperitoneal injection of D-luciferin. Body weight was also recorded. At the end of treatment, antitumor efficacy was further evaluated by H&E, Ki-67, and TUNEL staining. Major organs (heart, liver, spleen, lungs, and kidneys) of the mice were harvested for H&E staining to evaluate the in vivo biosafety.

### 4.13. Statistical Analysis

Data were displayed as mean ± SD (standard deviation). Statistical analysis was conducted through GraphPad Prism 9. Two-group comparisons were analyzed by two-tailed Student’s *t*-test; multiple comparisons were assessed by one-way or two-way ANOVA with Tukey’s test. *p*-values < 0.05 were regarded as statistically significant (* *p* < 0.05, ** *p* < 0.01, *** *p* < 0.001, **** *p* < 0.0001).

## Data Availability

No datasets were generated or analyzed during the current study. The data displayed in this study are available from the corresponding authors upon reasonable request.

## Supplementary Materials

The following supporting information can be downloaded at https://www.mdpi.com/article/10.3390/ijms27167475/s1.
