# Supplementary material for: Iron Oxide Nanozyme for Magnetothermal Activation of ER-TRPV1 Enables Efficient Intravesical Therapy of Bladder Cancer
Source: Int J Mol Sci. 2026 Aug 21;27(16):7475. doi: 10.3390/ijms27167475 (PMC13513725; doi:10.3390/ijms27167475)
Supplement: Supplementary file 1 [file ijms-27-07475-s001.zip › ijms-4429704-supplementary.pdf]

Supplementary Materials for

# Iron Oxide Nanozyme for Magnetothermal Activation of ER-TRPV1 Enables Efficient Intravesical Therapy of Bladder Cancer

Galong Li[1,2,#], Dongyan Li[1,3,#], Bin Lan[1], Yuanyuan Cheng[1], Hongxia Liang[1,3], Wenli Zhang[1], Xiaopan Xu[1,2,*], Hongbing Lu[1,*]

[1] School of Biomedical Engineering, Fourth Military Medical University, Xi'an, Shaanxi Province, 710032, China

[2] Medical Innovation Center, Fourth Military Medical University, Xi'an, Shaanxi Province, 710032, China

[3] College of Life Sciences, Northwest University, Xi'an 710069, China

[*] Correspondence: alexander-001@163.com (Xiaopan Xu); luhb@fmmu.edu.cn (Hongbing Lu)

[#] Galong Li and Dongyan Li contributed equally to this work.

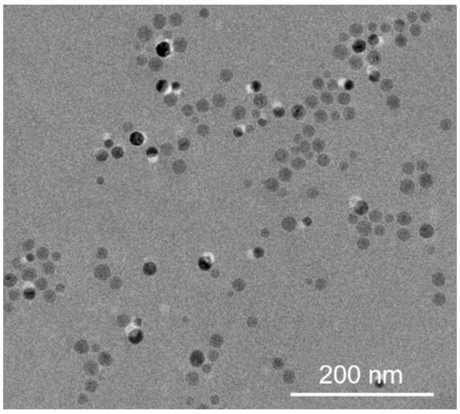


Figure S1. TEM image of anti-FGFR3-IONP.

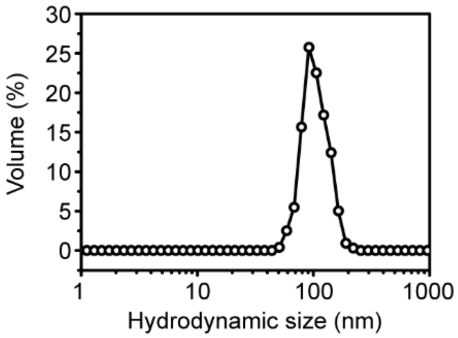


Figure S2. Hydrodynamic size of anti-FGFR3-IONP in artificial urine (containing (per liter): $CaCl_2$ 0.64 g, $MgCl_2$ 0.65 g, NaCl 4.60 g, $Na_2SO_4$ 2.30 g, KCl 1.60 g, $KH_2PO_4$ 2.80 g, $NH_4Cl$ 1.00 g, urea 25.0 g, and creatinine 1.10 g).

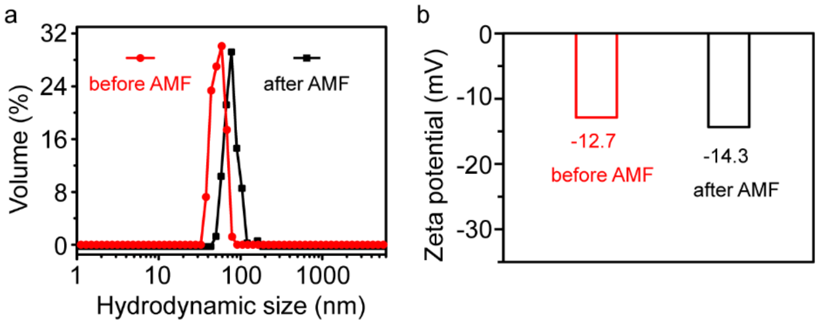


Figure S3. Characterization of anti-FGFR3-IONPs. (a) Hydrodynamic size distributions of anti-FGFR3-IONPs before and after AMF exposure. (b) Zeta potential of anti-FGFR3-IONPs before and after AMF exposure.

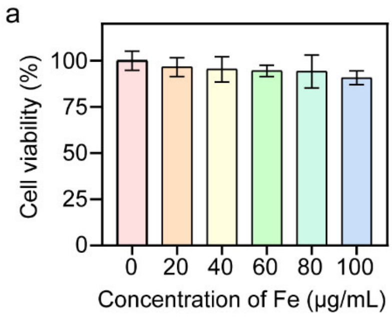


Figure S4. Cytotoxicity of IONPs at various Fe concentrations (from 0 to 100 μg/mL) in RT4 cells.

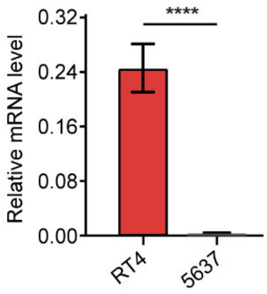


Figure S5. Relative mRNA level of FGFR3 expressed in RT4 and 5637 cells.

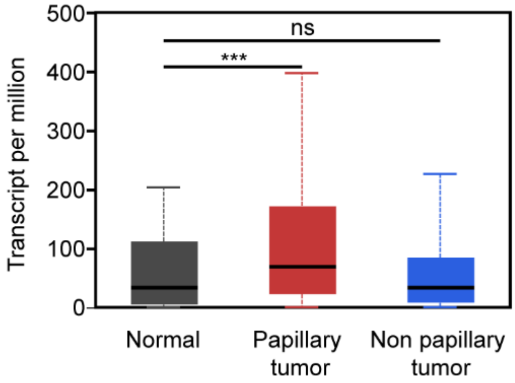


Figure S6. Differential expression of FGFR3 in bladder cancer based on histological subtypes. Box plots showing the transcripts per million (TPM) levels of FGFR3 in normal tissues (n = 19), papillary tumors (n = 132), and non-papillary tumors (n = 271). The data were obtained from the UALCAN database using TCGA samples. The central line represents the median, while the box limits indicate the upper and lower quartiles. ns, not significant; *$p$< 0.05; **$p$< 0.01; ***$p$< 0.001; ****$p$< 0.0001.

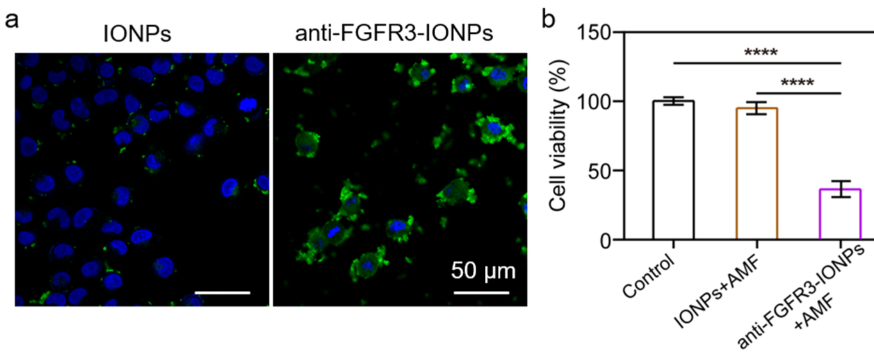


Figure S7. In vitro MHT effect. (a) CLSM images of RT4 cells after incubation with non-targeted IONPs and anti-FGFR3-IONP. Nuclei: Hoechst 33342 (blue); IONPs: FITC (green). Free nanoparticles were removed by washing the cells with a PBS buffer before imaging. (b) Cell viability of RT4 cells after treatment with non-targeted IONPs + AMF and anti-FGFR3-IONP + AMF. Data are displayed as mean ± SD (n=3). ns, not significant; *$p< 0.05$; **$p< 0.01$; ***$p< 0.001$; ****$p< 0.0001$.

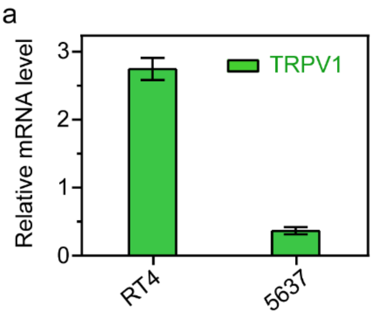


Figure S8. Relative expression levels of TRPV1 proteins in RT4 and 5637 cells.

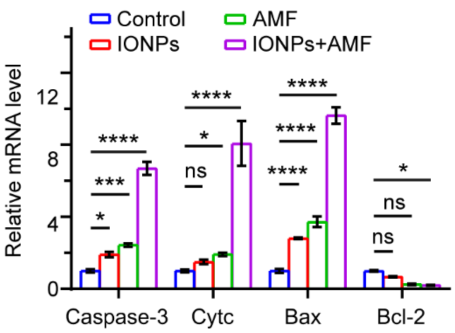


Figure S9. Relative mRNA expression levels of Caspase-3, Cytc, Bax, and Bcl-2 in RT4 cells. The data are displayed as mean ± SD (n=3). ns: no significant difference; *$p< 0.05$; **$p< 0.01$; ***$p< 0.001$; ****$p< 0.0001$.

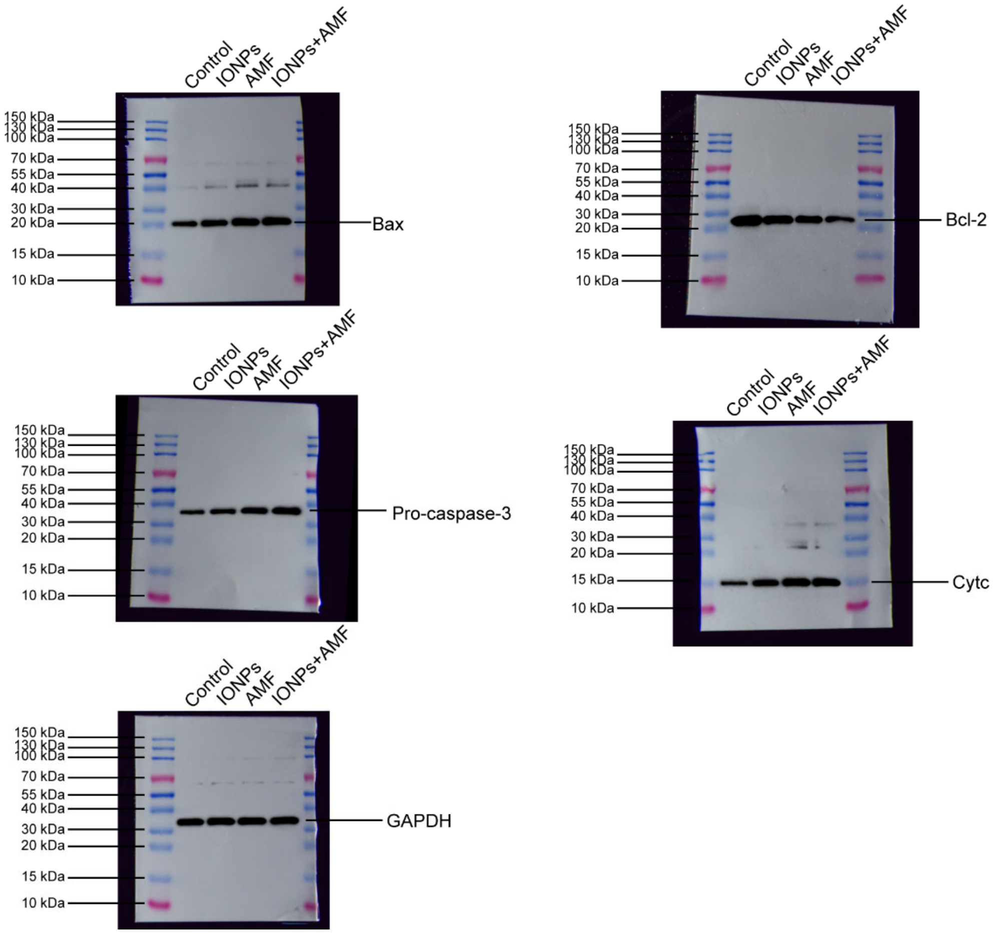


Figure S10. Western blot analysis of protein expression (Pro-caspase-3, Cytc, Bax, and Bcl-2) in RT4 cells after different treatments.

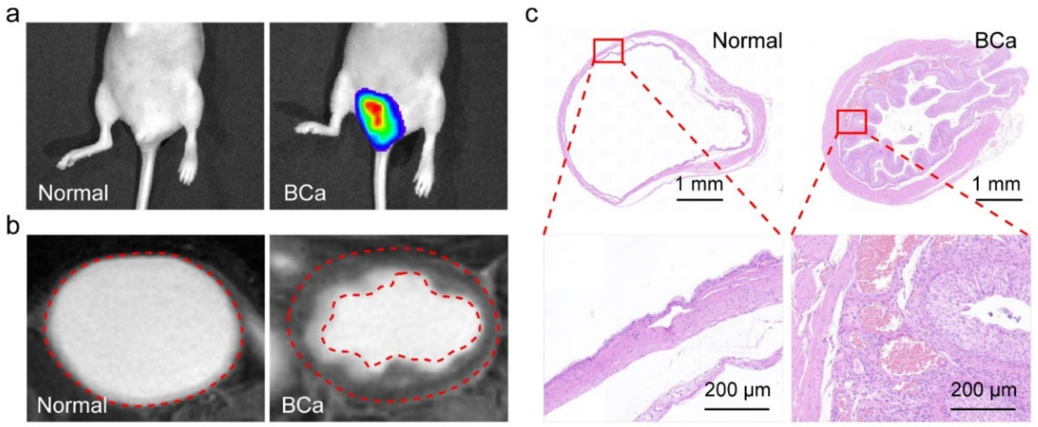


Figure S11. Characterization of an orthotopic BCa mouse model. (a) Representative bioluminescence images of normal and BCa mice on the 14th day after intravesical instillation of luciferase-labeled RT4 cells. (b) Ultrahigh-field MRI (9.4T) images of the bladder tissues in normal and BCa mice on the 14th day. (c) H&E-stained sections of bladder tissues from normal and BCa mice.

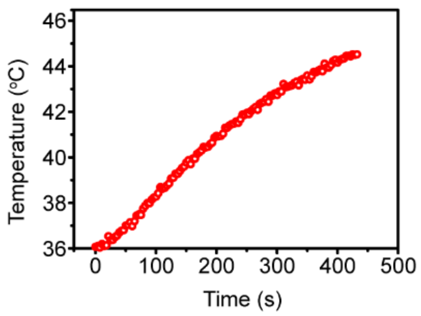


Figure S12. In vivo thermometry of the bladder in mice after intravesical instillation of anti-FGFR3-IONPs under AMF exposure.

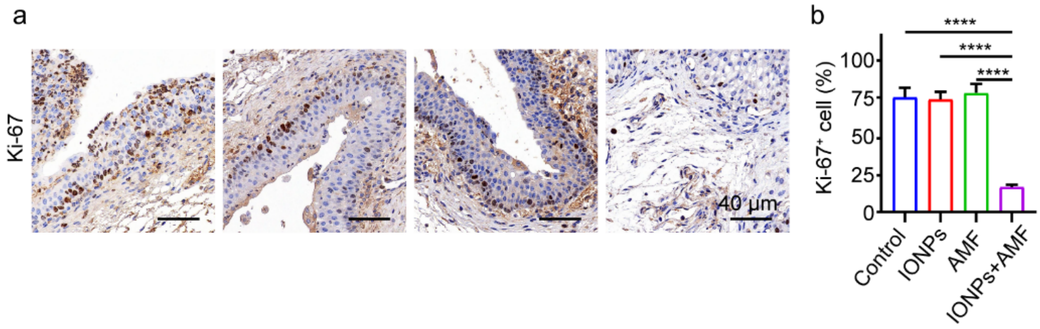


Figure S13. (a) Representative images of Ki-67 immunohistochemistry staining of BCa sections from mice after different treatments. (b) Quantification of Ki-67-positive cells in tumor tissues. The data are displayed as mean ± SD (n=3). ns: no significant difference; **p*< 0.05; ***p*< 0.01; ****p*< 0.001; *****p*< 0.0001.

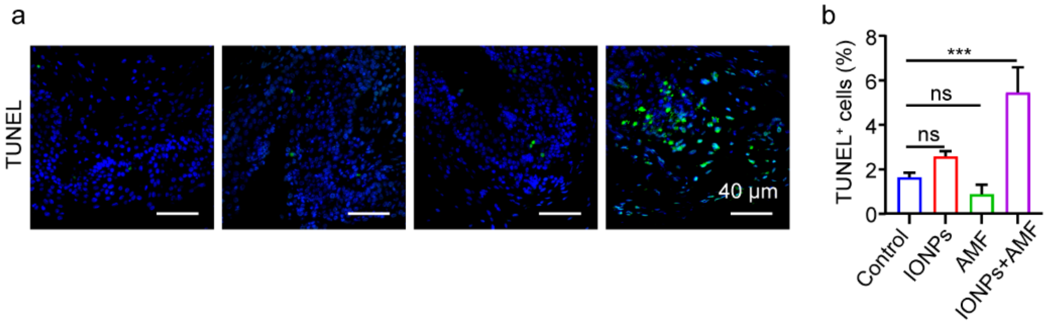


Figure S14. (a) Representative images of TUNEL immunofluorescence staining of BCa sections from mice after different treatments. (b) Quantification of TUNEL-positive cells in tumor tissues. The data are displayed as mean ± SD (n=3). ns: no significant difference; **p*< 0.05; ***p*< 0.01; ****p*< 0.001; *****p*< 0.0001.

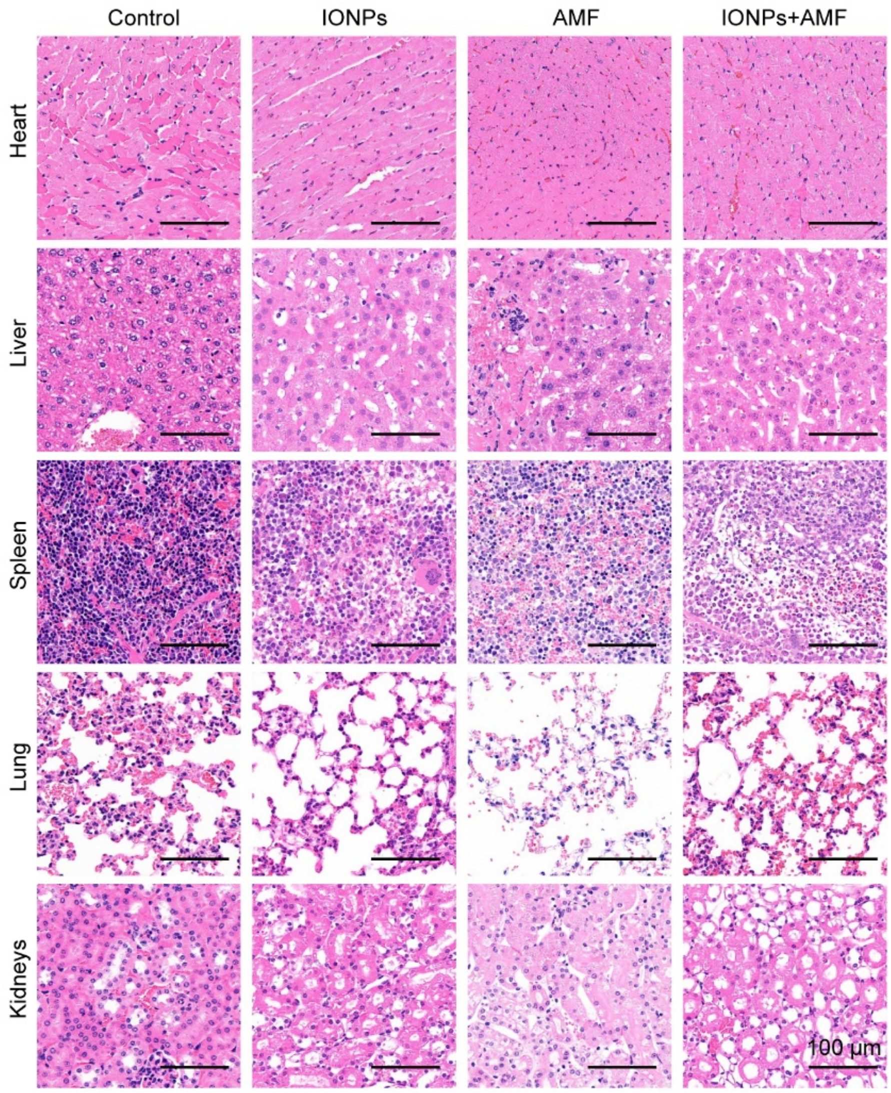


Figure S15. Representative H&E-stained histological sections of major organs (heart, liver, spleen, lungs, and kidneys) from mice in the control, IONPs, AMF, and IONPs+AMF groups. Scale bar: 100 μm. No notable signs of inflammation, tissue damage, or pathological abnormalities were observed. This suggests that intravesical perfusion of FGFR3-targeting IONPs demonstrates exceptional antitumor efficacy with minimal harm to normal tissues.

Table S1. List of primers used in RT-PCR.

| Name | Polarity | Sequence (5’- 3’) |
|---|---|---|
| human-actin | Forward | CAGATGTGGATCAGCAAGCAGGAG |
| | Reverse | GTCAAGAAAGGGTGTAACGCAACTAAG |
| human-FGFR3 | Forward | TGGGCTTCTTCCTGTTCATCC |
| | Reverse | CTTGTCAGTGGCATCGTCTTTC |
| human-TRPV1 | Forward | CCTGATGGCAAGGACGACTAC |
| | Reverse | GGGCAAAGTTCTTCCAGTGTC |
| M-GAPDH | Forward | CCTCGTCCCGTAGACAAAATG |
| | Reverse | TGAGGTCAATGAAGGGGTCGT |
| M-Casp3（2） | Forward | TGGAATGTCATCTCGCTCTGGT |
| | Reverse | GAAGAGTTTCGGCTTTCCAGTC |
| M-Cytc | Forward | CCAACAAGAACAAAGGCATCAC |
| | Reverse | CTGCCCTTTCTCCCTTCTTCTTA |
| M-Bax | Forward | GCCTTTTTGCTACAGGGTTTCAT |
| | Reverse | TATTGCTGTCCAGTTCATCTCCA |
| M-Bcl-2 | Forward | TGACTTCTCTCGTCGCTACCGT |
| | Reverse | CCTGAAGAGTTCCTCCACCACC |
